# Knowledge and Practice Gaps in Anaemia Prevention and Management Among Patients and Health Providers in Northern Ghana: A Comparative Cross‐Sectional Study

**DOI:** 10.1002/hsr2.72307

**Published:** 2026-04-08

**Authors:** Ellen Afetorgbor Kafui, Joseph Biluo Ngendal, Konpuoda David, Gabriel Owusu, Joel Karikari Nyarkoh, Safianu Apalebilah, Henrietta Eshun, Ama Frimpomaa Oware, Emmanuel Jingbeja, David Mawutor Donkor, Patrick Adu, Joseph Boachie

**Affiliations:** ^1^ Department of Medical Laboratory Sciences, College of Health and Allied Sciences University of Cape Coast Cape Coast Central Region Ghana; ^2^ Department of Social Work and Sociology University of Ghana Legon Greater Accra Ghana; ^3^ Department of Health, Physical Education and Recreation University of Cape Coast Cape Coast Ghana; ^4^ Department of Medical Laboratory Science, Faculty of Health and Allied Sciences KAAF University Gomoa Fetteh Kakraba Central Region Ghana

**Keywords:** anaemia management, anaemia prevention, behavioral determinants, healthcare providers, health literacy, KAP (Knowledge, Attitudes, Practices), Northern Ghana, patients' knowledge, socioeconomic disparities

## Abstract

**Background:**

Despite diverse national interventions, anaemia persists as a major public health burden in Ghana, with disproportionately high prevalence in the Northern Region. This persistence is driven by systemic inequities including limited healthcare access, socioeconomic deprivation, and cultural barriers that impede effective prevention and management. This comparative study assessed disparities in knowledge, attitudes, and practices (KAP) between patients and healthcare providers regarding anaemia prevention and management, highlighting critical gaps in current understanding.

**Aim:**

The study aimed to: (1) assess and compare KAP levels between patients and healthcare providers, (2) detect fundamental misconceptions affecting the management of anaemia, and (3) highlight any associations between anaemia prevalence and KAP variables among participants.

**Methods:**

This cross‐sectional comparative study was conducted across three healthcare facilities in Northern Ghana, involving 299 participants (141 healthcare providers and 158 patients). Structured questionnaires assessed KAP domains, and haemoglobin measurements determined anaemia status. *χ* ^2^ tests evaluated bivariate associations, while multivariable logistic regression identified independent predictors of anaemia, adjusting for sociodemographic and behavioral confounders. The comparative approach enabled identification of provider‐patient knowledge gaps to inform targeted interventions.

**Results:**

Healthcare providers demonstrated significantly higher knowledge levels, with 94.3% understanding anaemia's pathophysiological basis compared to 60.8% of patients (*p* = 0.004). Anaemia prevalence was significantly higher among patients (54.4%) compared to healthcare providers (33.3%; *χ*² = 13.45, *p* < 0.001). Key misconceptions persisted among patients, including beliefs that anaemia is contagious (8.9%) or spiritual (29.7%). Non‐health workers had lower adjusted odds of anaemia compared with health workers (aOR = 0.344, 95% CI: 0.184–0.641, *p* < 0.001). Participants with good attitudes and practices also had lower odds of anaemia (aOR = 0.549, 95% CI: 0.326–0.924, *p* = 0.024). Other variables including age, sex, marital status, education, region of residence, and history‐related factors were not significantly associated with anaemia in the adjusted model.

**Conclusions:**

Significant KAP disparities exist between healthcare providers and patients, with patient knowledge gaps and misconceptions substantially hindering anaemia prevention and management in Northern Ghana. Evidence‐based interventions should strengthen patient‐centered education programs, provide economic support for nutritional access, and implement continuous professional development for healthcare workers. Integration of these strategies into Ghana's Community‐Based Health Planning and Services (CHPS) program could significantly reduce the anaemia burden in resource‐limited settings.

## 1 Introduction

Anaemia remains a principal global health challenge with over 1.62 billion people affected worldwide. However, the higher impact persists among individuals in low‐ and middle‐income countries [1]. Over 60% anaemia prevalence is reported in vulnerable populations in sub‐Saharan Africa, exclusively among children below 5 years of age and pregnant mothers [2]. Similarly, high prevalence of anaemia is reported in Ghana, affecting more than 4‐in‐10 (40%) individuals. This is disproportionately persistent particularly in the Northern Region where the condition is exaggerated by disparities in healthcare access and socioeconomic limitations [3, 4]. Therefore, Ghana's Northern Region displays an overwhelming anaemia rate, with recent findings reporting more than 50% prevalence within certain districts [5]. In spite of diverse interventions to lessen anaemia burden in the past years, the rate is persistently high, suggesting the existence of crucial gaps in anaemia prevention and management strategies. Likewise, the effective management of anaemia is proposed to be hindered due to persistent gaps in knowledge, attitude and practices (KAP) among patients and healthcare providers [6].

In critically assessing the aetiology of anaemia in this region, studies have reported multifaceted factors including genetic conditions [7], infections of malaria [8] and helminths [9], as well as nutritional (iron) deficiencies, which is assigned the principal cause of anaemia [10]. These multifaceted factors pose exceptional challenges of anaemia management in resource‐limited settings. In addition, healthcare systems in northern Ghana are faced with other considerable impediments to effective anaemia control. This may include infrastructure limitations, medication stock outs, and workforce scarcities that compromise service delivery [11]. Some cultural beliefs and/or practices again complicate intervention efforts, as reported by a study highlighting misconceptions about anaemia's causes and treatment [6]. Therefore, patients, particularly in rural areas, often lack awareness of anaemia's causes and preventive measures [12].

The KAP of healthcare workers likewise epitomize another crucial dimension of anaemia management. Despite the fundamental role of healthcare workers in anaemia education, some studies have reported discrepancies in their knowledge and counselling practices [13]. Recent evidence reveal alarming gaps in health providers' knowledge regarding anaemia diagnosis and treatment protocols [14]. Likewise, varying counselling practices and insufficient follow‐up systems further compromise the effectiveness of interventions [15]. These provider‐level limitations interconnect with patient‐level factors to generate a multifaceted web of barriers against effective anaemia management.

The persistence of anaemia in Northern Ghana can be understood through multiple theoretical lenses. The Health Belief Model (HBM) suggests that individuals' health behaviors are influenced by perceived susceptibility, severity, benefits, and barriers [16]. In the context of anaemia, limited awareness of personal risk and misconceptions about causes may reduce perceived susceptibility, while economic constraints represent substantial barriers to preventive behaviors. The Social Determinants of Health (SDH) framework further illuminates how structural inequities, including poverty, food insecurity, limited education, and inadequate healthcare infrastructure, create conditions that perpetuate anaemia [17]. Additionally, Andersen's Behavioral Model of Health Services Use emphasizes the interplay between predisposing factors (sociodemographic characteristics, health beliefs), enabling factors (economic resources, healthcare access), and need factors (perceived and evaluated health status) in determining health service utilization [18]. These frameworks collectively suggest that addressing anaemia requires multi‐level interventions targeting individual knowledge, community resources, and health system capacity.

Recent global evidence highlights the persistent burden of anaemia. The WHO Global Anaemia Report 2024 indicates that 33% of women of reproductive age and 37% of pregnant women globally are anaemic, with the highest prevalence in sub‐Saharan Africa [19]. UNICEF's 2023 State of the World's Children report highlights that anaemia affects cognitive development and economic productivity, perpetuating intergenerational poverty cycles [20]. The Global Burden of Disease (GBD) 2019 study identified anaemia as a leading cause of years lived with disability (YLDs) in West Africa, with dietary iron deficiency accounting for over 50% of cases [21]. Despite this evidence, few empirical studies have employed a comparative design to assess provider‐patient KAP disparities using both biochemical markers (haemoglobin levels) and behavioral indicators in resource‐limited settings. This gap is particularly pronounced in Northern Ghana, where cultural, economic, and health system factors intersect to create unique challenges for anaemia management.

Although several anaemia‐related studies have explored its prevalence and pathogenesis, limited studies have thoroughly compared KAP between healthcare providers and patients. This suggests a concerning knowledge gap, given their intersecting roles contributing to the effective management of anaemia [22]. Assessing these disparities is critical for the development of targeted interventions that address both supply‐side (provider) and demand‐side (patient) barriers. In addition, northern Ghana's unique sociocultural context requires localized evidence to inform interventions. Evidence from studies in other regions may not be representative of this area's distinct challenges, comprising limited to seasonal food insecurity, high malaria transmission, and deeply rooted cultural beliefs about illness [23]. The current study therefore, provides context‐specific data to guide culturally appropriate solutions. The study compares KAP between healthcare providers and patients in Northern Ghana, highlighting critical gaps to inform policy.

This study was guided by the following hypotheses: (1) Healthcare providers have significantly higher knowledge and more favorable attitudes toward anaemia prevention compared to patients; (2) Significant KAP disparities exist between providers and patients that contribute to suboptimal anaemia management; (3) Higher knowledge and better practices are independently associated with lower odds of anaemia, after adjusting for sociodemographic and economic confounders; and (4) Misconceptions about anaemia etiology and management are more prevalent among patients than providers and are associated with increased anaemia risk.

## 2 Materials and Methods

### 2.1 Study Design

The current study adopted a comparative cross‐sectional design to assess the KAP about anaemia management and/or prevention between patients and healthcare providers in the Northern Region of Ghana. This design was primarily adopted as it enhanced the acquisition of concurrent data from participants in different healthcare facilities in this setting. Using this approach, the current study focused on achieving key objectives comprising: (1) assessment and comparison of KAP levels between patients and healthcare providers, (2) identification of fundamental misconceptions affecting the management of anaemia, and (3) determination of any associations between anaemia prevalence and KAP variables among participants.

Data collection was conducted during the dry season (November 2022 to February 2023) to minimize seasonal confounding from malaria transmission, which peaks during the rainy season (May to October) in Northern Ghana. This timing reduced the potential influence of seasonal malaria‐related anaemia on our prevalence estimates. However, we acknowledge that dry season food insecurity may have influenced dietary practices and nutritional anaemia. Seasonal variation was not explicitly controlled in the analysis but is acknowledged as a limitation.

### 2.2 Study Setting

This study was carried out in Northern Ghana, noted for its geographical uniqueness, which encounters some of the nation's utmost burdens of anaemia. The study was expressly executed across three designated healthcare facilities representing a continuum of healthcare delivery in this resource‐limited setting:

*Bawku Urban West Health Centre (Upper East Region)* **:** This is a primary‐level facility serving an approximate population of 40,000 in Bawku Municipality. This urban centre handles approximately 40–60 outpatient visits daily, with particular emphasis on maternal and child health services.

*St. Gregory Health Centre (Upper West Region)*: The facility is located in the rural Nanvilli Township near the Black Volta River. The facility records about 10–30 daily patient encounters. The services provided include maternity care, outpatient services as well as laboratory services.

*ECG Hospital, Kpandai (Northern Region):* This is a district‐level referral hospital serving approximately 100,000 residents across Kpandai and surrounding districts. The centre handles approximately 183 outpatient visits daily. As one of only two comprehensive facilities in the district, it manages complex anaemia cases including those complicated by sickle cell disease and severe malaria.

This setting was purposely selected to provide insights into anaemia management under real‐world constraints as encountered by many sub‐Saharan health systems. The inclusion of health facilities at different service levels was to enable the assessment of how healthcare delivery variations influence KAP regarding anaemia management.

These three facilities were purposively selected to represent the geographic, demographic, and socioeconomic diversity of Northern Ghana. The Upper East Region (Bawku Urban West Health Centre) is characterized by high population density, urban poverty, and proximity to international borders, with a predominantly Kusaal‐speaking population engaged in small‐scale trading and subsistence farming. The Upper West Region (St. Gregory Health Centre) represents rural, agrarian communities with limited infrastructure and seasonal food insecurity, serving primarily Dagaaba‐speaking populations. The Northern Region (ECG Hospital, Kpandai) bridges rural and peri‐urban contexts, serving Konkomba and Nanumba ethnic groups with mixed livelihoods including farming and fishing. Collectively, these sites capture the heterogeneity of Northern Ghana's healthcare landscape, including variations in facility capacity, patient demographics, disease burden (malaria, malnutrition), and cultural health beliefs. According to the 2021 Population and Housing Census, these regions share similar socioeconomic indicators: poverty rates exceeding 50%, literacy rates below 40%, and limited access to improved water and sanitation [24].

### 2.3 Study Population and Sampling

The current study utilized a dual‐population context to assess anaemia‐related KAP among healthcare providers and adult patients in northern Ghana. The healthcare provider group (*n* = 150) comprised healthcare personnel including physicians, nurse‐midwives, nutrition officers, and medical laboratory personnel. The patient population (*n* = 149) constituted exclusively patients aged 18 years or older. The study excluded critically ill patients and individuals with cognitive impairments that could compromise informed consent or survey participation. A multi‐stage sampling strategy was utilized to ensure population representativeness across the study sites. At the healthcare facility level, participants were allocated proportionally by caseload, with ECG Hospital contributing 70 healthcare providers and 70 patients, Bawku Health Centre 45 healthcare providers and 45 patients, and St. Gregory Health Centre 35 healthcare providers and 34 patients. For healthcare provider recruitment, we conducted comprehensive enumeration of eligible staff followed by stratified random sampling according to professional category (20% doctors, 50% nurses, 30% other clinical staff) and work experience (stratified at 5‐year intervals). Likewise, the patient recruitment was done by utilizing systematic sampling of outpatients and maternity patients, with every third eligible patient invited sequentially for participation. This comprehensive sampling methodology ensured the study population adequately represented the spectrum of healthcare providers and patients in northern Ghana's clinical settings.

### 2.4 Population and Sampling Frame

*Healthcare Providers:* The sampling frame comprised all healthcare workers (physicians, nurses, midwives, physician assistants, and laboratory scientists) actively employed at the three study facilities during the data collection period. The population frame included 187 eligible providers across the three sites.

*Patients:* The sampling frame included all adult outpatients (aged ≥ 18 years) attending the selected facilities during the study period, excluding emergency cases and critically ill patients.

### 2.5 Sample Size

Sample size was calculated using Cochran's formula for finite populations [25]:

n=(Z²×p×q)/d²

Where:

- *n* = required sample size
- Z = Z‐score corresponding to 95% confidence level (1.96)
- *p* = estimated proportion of anaemia in Northern Ghana (0.54, based on previous studies)
- *q* = 1 ‐ *p* = 0.46
- *d *= desired precision (margin of error = 0.05)

Initial calculation: *n* = (1.96² × 0.54 × 0.46)/0.05² *n *= (3.8416 × 0.2484)/0.0025 *n* = 0.954/0.0025 *n* = 381.6 ≈ 382.

Adjusting for finite population (*N* = 187 for healthcare providers): *n*_adjusted = *n*/(1 + (*n* − 1)/N) *n*_adjusted = 382/(1 + 381/187) *n*_adjusted = 382/3.04 *n*_adjusted = 125.7 ≈ 126.

To account for potential non‐response and incomplete data, we applied a design effect of 1.5: *n*_final = 126 × 1.5 = 189.

A total of 299 participants (141 health workers and 158 non‐health workers) were included in the final analysis. This sample size was considered adequate to support the statistical analyses performed in the study. Post‐hoc power analysis confirmed adequate statistical power (≥ 0.80) for detecting significant differences in KAP scores and anaemia prevalence between groups (detailed in Statistical Analysis section).

### 2.6 Response Rate and Non‐Participation

Of 195 healthcare providers approached, 150 participated (response rate: 76.9%). Reasons for non‐participation included: time constraints during work hours (*n* = 28, 14.4%), declined consent (*n* = 12, 6.2%), and did not meet inclusion criteria due to recent employment (< 6 months, *n* = 5, 2.6%).

Of 198 patients approached, 149 participated (response rate: 75.3%). Reasons for non‐participation included: declined consent (*n* = 31, 15.7%), time constraints (*n* = 12, 6.1%), and did not meet inclusion criteria (pregnancy, *n* = 4; recent blood transfusion, *n* = 2).

### 2.7 Design Effect Justification

The design effect (DEFF) of 1.5 was applied to account for potential clustering of responses within facilities and reduced precision compared to simple random sampling. This conservative estimate is consistent with WHO recommendations for health facility‐based surveys in resource‐limited settings. The design effect accounts for intra‐cluster correlation (ICC) arising from shared facility characteristics, provider training, and community health beliefs. While we did not calculate ICC empirically prior to data collection, a DEFF of 1.5 is standard for facility‐based studies with 3–5 clusters [26].

### 2.8 Cluster Effects and Regression Adjustment

To address potential cluster effects in our regression analyses, we conducted sensitivity analyses using generalized estimating equations (GEE) with robust standard errors, specifying facility as the clustering variable. Results were consistent with standard logistic regression, suggesting minimal cluster effects. However, we acknowledge that the small number of clusters (*n *= 3) limits the precision of cluster‐adjusted estimates. Standard logistic regression results are presented in the main analysis, with GEE results provided in Supporting Information S1: Table S2 for transparency.

## 3 Inclusion and Exclusion Criteria

### 3.1 Inclusion Criteria

#### 3.1.1 Healthcare Providers

- Currently employed at one of the three study facilities.
- Minimum of 6 months' clinical experience.
- Directly involved in patient care (clinical, laboratory, or counseling roles).
- Willing to provide informed consent and participate in both questionnaire and haemoglobin testing.

#### 3.1.2 Patients

- Age ≥ 18 years.
- Attending outpatient services at one of the three study facilities.
- Resident in Northern Ghana for at least 6 months.
- Willing to provide informed consent and participate in both questionnaire and haemoglobin testing.

### 3.2 Exclusion Criteria

#### 3.2.1 Both Groups

- Individuals with known haematological malignancies or chronic diseases affecting haemoglobin levels (e.g., chronic kidney disease, sickle cell disease).
- Recent blood transfusion (within 3 months).
- Pregnancy (for female participants, due to physiological haemodilution).
- Inability to provide informed consent due to cognitive impairment.
- Emergency or critically ill patients requiring immediate medical attention.

## 4 Recruitment Method

Healthcare providers were recruited through facility registers and staff meetings. Trained research assistants approached eligible providers during work hours, explained the study objectives, and invited voluntary participation.

Patients were recruited through systematic sampling at outpatient departments. Every third adult patient presenting to the outpatient clinic was approached by trained research assistants, screened for eligibility, and invited to participate. This approach ensured representativeness across different days of the week and times of day.

## 5 Data Collection and Laboratory Procedures

### 5.1 Quality Control and Bias Minimization

Several measures were implemented to ensure data quality and minimize bias:

*Interviewer Training:* Six research assistants (three per region) received intensive 2‐day training on questionnaire administration, ethical conduct, and standardized interview techniques. Training included role‐playing exercises, practice interviews, and inter‐rater reliability assessments. Research assistants were blinded to the study hypotheses to minimize expectation bias.

*Standardization:* All interviews were conducted using identical, structured questionnaires with standardized wording and skip patterns. Research assistants used neutral probing techniques and avoided leading questions.

*Prevention of Respondent Duplication:* Each participant was assigned a unique identifier upon enrollment. A master registry was maintained at each facility, recording participant names, contact information, and date of enrollment. Research assistants cross‐checked this registry before each interview to prevent duplicate enrollment. Additionally, participants were asked at the beginning of each interview whether they had previously participated in the study.

*Social Desirability Bias:* To minimize social desirability bias, participants were assured of confidentiality and anonymity. Questionnaires were administered in private settings, and participants were encouraged to answer honestly without fear of judgment. Questions were framed neutrally, and response options included “don't know” to reduce pressure to provide socially acceptable answers.

*Data Verification:* Daily data quality checks were conducted by the principal investigators, including review of completed questionnaires for completeness, consistency, and logical errors. Any discrepancies were resolved through re‐contact with participants (when possible) or flagged as missing data.

#### 5.1.1 Haemoglobin (Hb) Screening

All the 299 enrolled participants (150 healthcare providers and 149 patients) underwent an objective haemoglobin measurement to determine their anaemia status. Haemoglobin (Hb) levels were measured using the HemoCue Hb 301 system (HemoCue AB, Ängelholm, Sweden), a point‐of‐care device validated for use in field settings [27]. The HemoCue Hb 301 employs a modified azide methemoglobin method with accuracy comparable to laboratory reference methods (correlation *r* > 0.95) [28].

### 5.2 Calibration and Quality Control

*Daily Calibration:* Each HemoCue device was calibrated daily before use according to manufacturer specifications. Self‐test procedures were performed using HemoCue control cuvettes (HemoCue Control High and Low) to verify device accuracy. Devices were only used if control values fell within acceptable ranges (±5% of target values).

*Environmental Controls:* All measurements were conducted in shaded, temperature‐controlled environments (20°C–25°C) to minimize temperature‐related measurement errors. Cuvettes were stored at room temperature and protected from direct sunlight and humidity.

*Instrument Maintenance:* Devices were cleaned daily using lint‐free cloths. Optical surfaces were inspected for dust or debris. Devices were serviced and recalibrated by certified technicians every 3 months during the study period.

*Duplicate Measurements:* For quality assurance, 10% of participants (*n* = 30) underwent duplicate Hb measurements by different research assistants using different HemoCue devices. The mean difference between duplicate measurements was 0.3 g/dL (95% CI: 0.1–0.5 g/dL), within acceptable limits.

### 5.3 Blood Sampling Procedure

Capillary blood samples were obtained through finger prick using sterile, single‐use lancets. The first drop of blood was discarded to avoid tissue fluid contamination. The second drop was collected directly into the HemoCue microcuvette, avoiding air bubbles. Measurements were taken immediately (within 10 s) to prevent clotting.

### 5.4 Anaemia Classification

Anaemia was defined according to WHO criteria [29]:

- Non‐pregnant women: Hb < 12.0 g/dL.
- Men: Hb < 13.0 g/dL.
- Pregnant women: Hb < 11.0 g/dL (excluded from main analysis).
- Severity classification:
- Mild anaemia: Hb 10.0–11.9 g/dL (women), 10.0–12.9 g/dL (men).
- Moderate anaemia: Hb 7.0–9.9 g/dL.
- Severe anaemia: Hb < 7.0 g/dL.

### 5.5 Sensitivity Analysis

Given potential measurement variability (±0.5 g/dL), we conducted sensitivity analyses by reclassifying anaemia status using adjusted thresholds (±0.5 g/dL). Results showed minimal impact on prevalence estimates (difference < 3%), suggesting robust classification.

### 5.6 Data Collection Tool

The study employed a structured questionnaire specifically designed to assess knowledge, attitudes, and practices (KAP) related to anaemia management among healthcare providers and patients in northern Ghana. The instrument was developed through an iterative process incorporating expert consultation, literature review [30], and pilot testing to ensure content validity and cultural appropriateness for the target populations.

The KAP domains were operationalized as follows: the knowledge domain assessed factual understanding of anaemia causes, symptoms, consequences, and prevention; the misconceptions domain captured culturally rooted or erroneous beliefs regarding anaemia (e.g., spiritual causation); and the attitudes/practices domain evaluated health‐seeking behaviours, dietary practices, and preventive actions related to anaemia management.

The structured questionnaire consisted of five sections; socio‐demographic information (age, gender, education, occupation, health history), knowledge assessment (causes, symptoms, and risks of anaemia), misconceptions (cultural or erroneous beliefs about anaemia), prevention and management strategies (diet, supplements, medical interventions), and attitudes and practices (health‐seeking behaviour, dietary habits, and adherence to preventive measures). The tool employed closed‐ended questions to ensure quantifiable data.

For healthcare providers, the questionnaire was available in English via electronic format, while patient versions were translated and back‐translated into local languages (Dagbani and Mampruli) and administered in interview format. The complete instrument required approximately 20 min for completion, with pilot testing confirming acceptable respondent burden. Validation processes included expert review by haematologists and public health specialists, cognitive testing with target populations, and assessment of internal consistency (Cronbach's *α* = 0.91, 0.70, and, 0.80, for Knowledge, Misconception, and, Attitudes and Practices domains, respectively). The structure facilitated comprehensive KAP assessment while accommodating the literacy and cultural contexts of diverse participants in this resource‐limited setting.

For scoring, each correct knowledge response was assigned a score of 1, while incorrect and “not sure” responses were scored 0. Misconception items were reverse‐coded so that higher scores indicated fewer misconceptions. Attitudes and practices items were scored based on alignment with recommended anaemia prevention behaviours, with preventive practices receiving a score of 1 and non‐preventive or non‐adherent responses scored 0.

Composite scores for each domain were calculated by summing the individual item scores. The domain score distributions were then examined, and domain means were computed. The observed mean scores were 9.21 ± 1.00 for the knowledge domain, 6.04 ± 1.30 for the misconception's domain, and 5.89 ± 1.12 for the attitudes and practices domain. These mean scores were used as empirically driven thresholds to dichotomise participants into higher versus lower scoring groups.

During pilot testing, the instrument was administered to 30 participants (approximately 10% of the projected sample) drawn from a neighbouring community with similar sociodemographic characteristics. Acceptability and respondent burden were evaluated based on average time to completion, participant comprehension as judged by interviewer feedback, and item non‐response rates. The mean completion time was approximately 18 min, most respondents (> 95%) reported that questions were easy to understand, and overall item non‐response was low (< 3% per item), supporting the acceptability and clarity of the questionnaire.

### 5.7 Ethical Consideration

The study followed the ethical protocols approved by the Institutional Review Board (IRB) of the University of Cape Coast (Approval Numbe: UCCIRB/CHAS/2023/02). Likewise, informed consent was acquired from all recruited participants prior to their involvement in the current study. The participants were adequately informed about the study's aims and objectives, highlighting their exclusive right to withdraw from the study at any time. No personally identifiable information was collected or disclosed in the study. Data were anonymized and securely stored, accessible only to the research team. The questionnaires and haemoglobin (Hb) measurements were coded to prevent connection to individual identities. All data were double‐checked and kept in password‐protected files.

Written informed consent was obtained from all participants prior to enrollment. For literate participants, consent forms were provided in English or translated versions (Dagbani, Mampruli), and participants were given adequate time to read and ask questions before signing.

For illiterate participants (approximately 35% of patient sample), the following modified consent procedure was implemented in accordance with Ghana Health Service guidelines:

1. Verbal Explanation: Trained research assistants provided detailed verbal explanation of study purpose, procedures, risks, benefits, and rights in the participant's preferred language.
2. Witness Requirement: An impartial witness (not a study team member), typically a family member or community member trusted by the participant, was present during the consent process.
3. Thumbprint Consent: Illiterate participants provided thumbprint consent on the consent form in the presence of the witness, who also signed to attest that the information was accurately conveyed and understood.
4. Comprehension Assessment: Research assistants asked participants to explain key study elements in their own words to confirm understanding before obtaining consent.

### 5.8 Data Confidentiality and Privacy

Multiple measures ensured participant confidentiality:

1. De‐identification: Each participant was assigned a unique identification number. Data collection forms and laboratory specimens were labeled only with ID numbers, not names.
2. Secure Storage: Paper consent forms (containing identifiable information) were stored separately from de‐identified data forms in locked cabinets accessible only to principal investigators. Electronic data files were password‐protected and stored on encrypted devices.
3. Limited Access: Only authorized research team members had access to identifiable data. Data analysts received fully de‐identified datasets.
4. Private Interview Settings: All interviews and haemoglobin testing were conducted in private spaces (consultation rooms, private offices) to ensure confidentiality and minimize social desirability bias.
5. Anonymized Reporting: All published results present aggregate data only. No individual participants can be identified from reported findings.

### 5.9 Protections for Vulnerable Participants and Clinical Referrals

Pregnant women were excluded from the main analysis due to different haemoglobin thresholds for anaemia diagnosis and potential confounding from physiological haemodilution. However, pregnant women who consented to participate (*n* = 8) were tested for anaemia and received appropriate counseling and referrals but were not included in the analytical sample.

All participants found to have anaemia during the study were provided with:

1. Immediate Counseling: Trained research assistants provided culturally appropriate counseling on anaemia causes, consequences, and management strategies.
2. Clinical Referral: Participants with moderate or severe anaemia (Hb < 10.0 g/dL) were immediately referred to facility clinicians for further evaluation and treatment. Referral letters documented haemoglobin levels and recommended follow‐up care.
3. Nutritional Education: All anaemic participants received individualized nutritional counseling and printed educational materials (in local languages) on iron‐rich foods and dietary diversification strategies.
4. Follow‐Up: Contact information was collected (with consent) to enable follow‐up communication. Research assistants contacted referred participants 2 weeks post‐referral to confirm clinic attendance and treatment initiation.

Participants with severe anaemia (Hb < 7.0 g/dL, *n* = 4) received priority referrals with direct communication to facility physicians to ensure immediate clinical attention. These participants were accompanied to the consultation room to ensure timely care.

No financial incentives were provided for participation; however, all testing and counseling services were provided free of charge.

### 5.10 Data Availability Statement

The de‐identified dataset supporting the findings of this study is available from the corresponding authors upon reasonable request, subject to approval from the University of Cape Coast Institutional Review Board. Requests should include a brief research proposal describing the intended use of the data and evidence of ethical approval from the requester's institution. The study questionnaire, data collection forms, and statistical analysis code are available in the Supporting Information S1 (Appendices A–C). Individual participant data will not be shared to protect participant privacy, but aggregate data and summary statistics can be provided for meta‐analyses or systematic reviews upon request.

### 5.11 Statistical Analysis

The study utilized a quantitative analytical approach to comparatively assess the KAP of healthcare providers and patients about anaemia management. All data, constituting the questionnaire responses and haemoglobin measurements, were entered into Microsoft Excel 2016 for initial coding and cleaning. Likewise, comprehensive crosschecking of the data was done before analysis using IBM SPSS Version 26. Both descriptive statistics and comparative analyses using Chi‐square (*χ*²) and Fisher's Exact tests were utilised for association assessments.

Normality of continuous variables was assessed using the Shapiro–Wilk test in conjunction with visual inspection of Q–Q plots. The test indicated no significant deviation from normality (*p* > 0.05) for haemoglobin levels. Homogeneity of variances across comparison groups was evaluated using Levene's test, which also showed no statistically significant differences in variances (*p* > 0.05).

To identify predictors of anaemia prevalence while adjusting for potential confounders such as education and occupation, multivariable logistic regression was performed. The results were reported as adjusted odds ratios (AORs) with 95% confidence intervals (CIs). Statistical significance was determined using a threshold *p*‐value of less than 0.05. Variables included in the multivariable models were selected a priori based on theoretical relevance and empirical evidence from the literature, and all potential variables were assessed for multicollinearity before inclusion. Model fit was assessed using the Hosmer–Lemeshow test, and multicollinearity was evaluated using Variance Inflation Factors (VIF < 10). Additionally, linear regression with robust standard errors was performed to assess predictors of continuous haemoglobin (Hb) levels. Results were presented as β‐coefficients with 95% CI.

Post hoc power analysis was conducted for the primary statistical tests. For the multivariable logistic regression model, adequacy of power was assessed using the events‐per‐variable criterion, yielding an EPV of 9.5. For the chi‐square analyses, post‐hoc power was estimated based on observed effect sizes and sample size. While several comparisons demonstrated adequate power (≥ 0.80), some tests yielded lower power, reflecting smaller effect sizes or limited cell frequencies.

Sociodemographic, knowledge, misconception, attitude, and practice variables were included in the analysis based on theoretical relevance. Anaemia status was the outcome variable. All predictors were entered simultaneously into the multivariable logistic regression model. Knowledge, misconception, attitude, and practice variables were derived from composite scores. Educational level was recategorized by merging non‐formal and basic education into a single category to address sparse cell counts. Smoking status and religion were excluded from the multivariable analysis due to low observations in some categories that could compromise model stability.

## 6 Results

### 6.1 Sociodemographic Profile

Table 1 summarizes the socio‐demographic characteristics of the 299 study participants. The majority were older than 30 years (50.8%), followed by those aged 26–30 years (22.4%). Sex distribution was almost equal, with males at 50.8% and females at 49.2%. Most participants were married (51.2%) and had tertiary education (67.2%). Health workers comprised 47.2% of the sample, while non‐health workers accounted for 52.8%. Christianity was the predominant religion (67.7%), and the participants were fairly distributed across the Northern (33.4%), Upper East (34.9%), and Upper West (31.8%) regions. A minority reported chronic health conditions (11.7%), anaemia history in the past 3 years (17.4%), and blood transfusion (18.1%), or supplement use (46.2%). These details provide critical background for understanding patterns in anaemia knowledge, attitude, and practice.

**Table 1:** Sociodemographic characteristics of study participants (*n* = 299).

Variable	Frequency (%)
Age (years)	
Below 20	16 (5.4)
20–25	64 (21.4)
26–30	67 (22.4)
Above 30	152 (50.8)
Sex	
Male	152 (50.8)
Female	147 (49.2)
Marital status	
Single	146 (48.8)
Married	153 (51.2)
Educational level	
Non‐formal	49 (16.4)
Basic	6 (2.0)
Secondary	43 (14.4)
Tertiary	201 (67.2)
Occupation	
Health worker	141 (47.2)
Non‐health worker	158 (52.8)
Religion	
Christianity	202 (67.6)
Islam	90 (30.1)
Traditional	7 (2.3)
Region of residence	
Northern	100 (33.4)
Upper East	104 (34.8)
Upper West	95 (31.8)
Chronic health status	
Yes	35 (11.7)
No	264 (88.3)
History of anaemia in the past 3 years	
Yes	52 (17.4)
No	247 (82.6)
Do you smoke	
Yes	7 (2.3)
No	292 (97.7)
Do you take alcohol	
Yes	47 (15.7)
No	252 (84.3)
History of blood transfusion	
Yes	54 (18.1)
No	245 (81.9)
Have you ever taken iron supplement?	
Yes	138 (46.2)
No	161 (53.8)
Hb (g/dL)	12.1 ± 1.98

### 6.2 Knowledge and Misconceptions of Participants

#### 6.2.1 A. Anaemia‐Related Knowledge of Participants

Table 2 presents the distribution of anaemia‐related knowledge across occupation groups. Several of the largest contrasts occurred in foundational knowledge items where health workers consistently showed higher proportions of correct responses. For instance, 94.3% of health workers correctly identified that anaemia reduces the blood's ability to carry oxygen compared with 60.8% of non‐health workers (*χ*² = 53.579, *p* < 0.001, Cramer's *V* = 0.423). Similarly, 97.2% of health workers recognized poor diet and malaria as causes of anaemia vs. 71.5% of non‐health workers (*χ*² = 40.562, *p* < 0.001, Cramer's *V *= 0.368). High contrasts also appeared for the understanding that anaemia can lead to death, with 99.3% of health workers and 82.9% of non‐health workers responding correctly (*χ*² = 23.562, *p*< 0.001, Cramer's *V* = 0.281). Supporting Information S1: Table S2 presents the correlation coefficients between haemoglobin concentration and the knowledge, misconception, and attitudes/practices scores. The correlations were minimal and not statistically significant, indicating that higher knowledge or more favourable attitudes alone were not directly associated with haemoglobin levels in this study.

**Table 2:** Knowledge of healthcare workers and patients on anaemia management, prevention, treatment, and misconceptions (*n* = 299).

	Total, *n* (%)	Occupation		Cramer's *V*	*χ*²	*p* value
Variables		Health worker (*n* = 141)	Non‐health worker (*n* = 158)			
*Knowledge of participants on Anaemia*						
Blood has a reduced ability to carry oxygen when anaemia is present						
Not sure	51 (17.0)	1 (0.7)	50 (31.6)	0.423	53.579	**< 0.001** ^**α**^
No	19 (6.4)	7 (5.0)	12 (7.6)			
Yes	229 (76.6)	133 (94.3)	96 (60.8)			
Dizziness and weakness may be some symptoms of anaemia						
Not sure	14 (4.7)	0 (0)	14 (8.9)	0.232	16.085	**< 0.001**
No	3 (1.0)	0 (0)	3 (1.9)			
Yes	282 (94.3)	141 (100.0)	141 (89.2)			
Intake of poor diet and malaria infection are some causes anaemia						
Not sure	43 (14.4)	1 (0.7)	42 (26.6)	0.368	40.562	**< 0.001** ^**α**^
No	6 (2.0)	3 (2.1)	3 (1.9)			
Yes	250 (83.6)	137 (97.2)	113 (71.5)			
When untreated, anaemia could lead to death						
Not sure	26 (8.7)	1 (0.7)	25 (15.8)	0.281	23.562	**< 0.001** ^**α**^
No	2 (0.7)	0 (0)	2 (1.3)			
Yes	271 (90.6)	140 (99.3)	131 (82.9)			
Anaemia could result in impaired development in children						
Not sure	57 (19.1)	5 (3.5)	52 (32.9)	0.382	43.603	**< 0.001** ^**α**^
No	9 (3.0)	3 (2.1)	6 (3.8)			
Yes	233 (77.9)	133 (94.4)	100 (63.3)			
Pregnant women are mostly at risk of anaemia						
Not sure	41 (13.7)	0 (0)	41 (25.9)	0.387	44.819	**< 0.001** ^**α**^
No	2 (0.7)	0 (0)	2 (1.3)			
Yes	256 (85.6)	141 (100.0)	115 (72.8)			
Vegetarians are mostly at risk of vitamin B12 deficiency anaemia						
Not sure	94 (31.4)	22 (15.6)	72 (45.6)	0.325	31.634	**< 0.001**
No	41 (13.7)	26 (18.4)	15 (9.5)			
Yes	164 (54.9)	93 (66.0)	71 (44.9)			
Excess intake of alcohol leads to anaemia						
Not sure	43 (14.4)	10 (7.1)	33 (20.9)	0.205	12.571	**0.002**
No	21 (7.0)	13 (9.2)	8 (5.0)			
Yes	235 (78.6)	118 (83.7)	117 (74.1)			
Iron deficiency can cause anaemia						
Not sure	52 (17.4)	0 (0)	52 (32.9)	0.442	58.525	**< 0.001** ^**α**^
No	7 (2.3)	2 (1.4)	5 (3.2)			
Yes	240 (80.3)	139 (98.6)	101 (63.9)			
Intake of non‐nutritious substances like clay can cause anaemia						
Not sure	69 (23.1)	8 (5.7)	61 (38.6)	0.409	49.968	**< 0.001**
No	24 (8.0)	9 (6.4)	15 (9.5)			
Yes	206 (68.9)	124 (87.9)	82 (51.9)			
*Misconceptions of participants*						
Anaemia is a spiritual condition						
Not sure	35 (11.7)	7 (5.0)	28 (17.7)	0.464	64.335	**< 0.001**
No	215 (71.9)	132 (93.6)	83 (52.5)			
Yes	49 (16.4)	2 (1.4)	47 (29.8)			
Anaemia is contagious or transmissible by body contact						
Not sure	33 (11.0)	2 (1.4)	31 (19.6)	0.328	32.129	**< 0.001**
No	248 (82.9)	135 (95.7)	113 (71.5)			
Yes	18 (6.1)	4 (2.8)	14 (8.9)			
Sickle cell anaemia affects Males more than females						
Not sure	92 (30.8)	22 (15.6)	70 (44.3)	0.335	33.603	**< 0.001**
No	191 (63.9)	114 (80.9)	77 (48.7)			
Yes	16 (5.3)	5 (3.5)	11 (7.0)			
Men are more prone to anaemia compared to women						
Not sure	79 (26.4)	10 (7.1)	69 (43.7)	0.476	67.757	**< 0.001**
No	206 (68.9)	130 (92.2)	76 (48.1)			
Yes	14 (4.7)	1 (0.7)	13 (8.2)			
Consistently eating thrice a day can prevent anaemia						
Not sure	44 (14.7)	11 (7.8)	33 (20.9)	0.385	44.281	**< 0.001**
No	162 (54.2)	105 (74.5)	57 (36.1)			
Yes	93 (31.1)	25 (17.7)	68 (43.0)			
Pregnant women who eat well do not require iron and folic acid supplements						
Not sure	62 (20.7)	9 (6.4)	53 (33.5)	0.487	70.777	**< 0.001**
No	177 (59.2)	119 (84.4)	58 (36.7)			
Yes	60 (20.1)	13 (9.2)	47 (29.8)			
Anaemia is a disorder of the heart						
Not sure	84 (28.1)	19 (13.5)	65 (41.1)	0.376	42.208	**< 0.001**
No	191 (63.9)	117 (83.0)	74 (46.8)			
Yes	24 (8.0)	5 (3.5)	19 (12.1)			

*Note:* α: Fisher's Exact Test. All *χ*² tests were conducted with degree of freedom (df) = 2. Values are shown as frequencies (*n*) and percentage (%). Percentages are column percentages. Total sample size = 299. *p*‐values < 0.05 are presented in boldface to indicate statistical significance. Misconception items were reverse‐coded; therefore, higher misconception scores represent fewer misconceptions and a more accurate understanding of anaemia.

#### 6.2.2 B. Misconceptions of Participants About Anaemia

The misconception items similarly showed notable contrasts, with several variables displaying high chi‐square values and large effect sizes. Among these, 93.6% of health workers rejected the claim that anaemia is a spiritual condition, compared with 52.5% of non‐health workers (*χ*² = 64.335, *p* < 0.001, Cramer's *V* = 0.464). A comparable pattern was seen for the belief that men are more prone to anaemia, which was rejected by 92.2% of health workers but only 48.1% of non‐health workers (*χ*² = 67.757, *p* < 0.001, Cramer's *V* = 0.476). Another strong contrast appeared for the misconception that pregnant women who eat well do not require supplements, where 84.4% of health workers disagreed compared with 36.7% of non‐health workers (*χ*² = 70.777, *p* < 0.001, Cramer's *V* = 0.487). These variables accounted for the largest occupation‐related differences in misconceptions recorded in the table.

### 6.3 Attitudes and Practices Towards Anaemia Prevention and Management

Table 3 compares attitudes and practices related to anaemia across occupation groups. Several items showed measurable contrasts, with higher proportions of health workers demonstrating favourable attitudes toward health‐seeking behaviours. For example, 87.9% of health workers reported that they would consult a physician for symptoms such as dizziness and shortness of breath compared with 70.3% of non‐health workers (*χ*² = 13.860, *p* < 0.001, Cramer's *V* = 0.215). A clear difference was also observed in seeking anaemia‐related health education, with 80.9% of health workers and 60.8% of non‐health workers responding positively (*χ*² = 14.387, *p* < 0.001, Cramer's *V* = 0.219). The strongest contrast in the table occurred for the belief that any health worker can prescribe anaemia medication, with 92.2% of health workers rejecting this statement compared with 63.3% of non‐health workers (*χ*² = 35.074, *p* < 0.001, Cramer's *V* = 0.342). Additional variables showed smaller contrasts but followed the general pattern of higher affirmative or corrective responses among health workers.

**Table 3:** Association between occupation and attitudes and practices towards anaemia prevention and management.

	Total, *n* (%)	Health worker (*n* = 141)	Non‐health worker (*n* = 158)	Cramer's *V*	*χ*²	*p* value
Do you consult a Physician when you have symptoms like dizziness and shortness of breath?				0.215	13.860	**< 0.001**
Yes	235 (78.6)	124 (87.9)	111 (70.3)			
No	64 (21.4)	17 (12.1)	47 (29.7)			
Do you seek health education on anaemia?				0.219	14.387	**< 0.001**
Yes	210 (70.2)	114 (80.9)	96 (60.8)			
No	89 (29.8)	27 (19.1)	62 (39.2)			
I prefer to eat a heavy meal once or twice a day due to financial issues.				0.215	13.834	**< 0.001**
Yes	140 (46.8)	50 (35.5)	90 (57.0)			
No	159 (53.2)	91 (64.5)	68 (43.0)			
Any health worker can prescribe anaemia medication for me				0.342	35.074	**< 0.001**
Yes	69 (23.1)	11 (7.8)	58 (36.7)			
No	230 (76.9)	130 (92.2)	100 (63.3)			
Do you think the food you eat has nothing to do with anaemia?				0.381	43.298	**< 0.001**
Yes	89 (29.8)	16 (11.3)	73 (46.2)			
No	210 (70.2)	125 (88.7)	85 (53.8)			
I can manage anaemia on my own without the help of a clinician				0.205	12.572	**< 0.001**
Yes	50 (16.7)	35 (24.8)	15 (9.5)			
No	249 (83.3)	106 (75.2)	143 (90.5)			
I'd rather eat vegetables than take supplements				0.045	0.613	0.487
Yes	154 (51.5)	76 (53.9)	78 (49.4)			
No	145 (48.5)	65 (46.1)	80 (50.6)			
Would you participate in pre‐marital counselling and testing before having children?				0.165	8.125	**0.006**
Yes	245 (81.9)	125 (88.7)	120 (75.9)			
No	54 (18.1)	16 (11.3)	38 (24.1)			
There's no need to follow up on my health once I've been prescribed medication				0.162	7.879	**0.007**
Yes	41 (13.7)	11 (7.8)	30 (19.0)			
No	258 (86.3)	130 (92.2)	128 (81.0)			
I don't see the importance of sleeping under insecticide treated bed net				0.000	0.000	> 0.99
Yes	36 (12.0)	17 (12.1)	19 (12.0)			
No	263 (88.0)	124 (87.9)	139 (88.0)			

*Note:* All *χ*² tests were conducted with degree of freedom (df) = 1.Bold text indicates statistically significant *p*‐values (*p* < 0.05).

Table 4 provides summary scores for knowledge, misconceptions, and attitudes/practices by occupation group. Health workers recorded higher mean (9.21 ± 1.03) knowledge scores and higher misconception scores, while attitudes/practices scores were broadly comparable between the groups.

**Table 4:** Summary of knowledge, misconception, and attitude scores by occupation.

Score Type	Health workers (*n* = 141)	Non‐health workers (*n* = 158)
Knowledge score	9.21 ± 1.03 (6–10)	6.75 ± 3.32 (0–10)
Misconception score	6.04 ± 1.30 (1–7)	3.41 ± 2.43 (0–7)
Attitudes and practices score	5.89 ± 1.31 (2–8)	5.63 ± 1.35 (0–10)

*Note:* Data is presented as Mean ± standard deviation (range).

**Figure 1:**
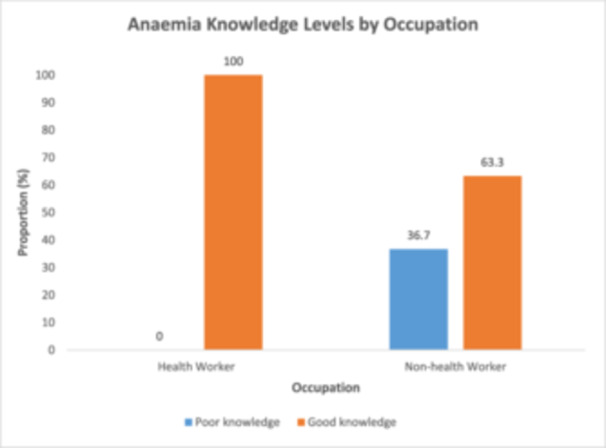
Distribution of knowledge categories by occupation.

Figure 1 shows the distribution of knowledge categories by occupation. All health workers were classified as having good knowledge (100%), with none falling into the poor knowledge category. Among non‐health workers, 63.3% were classified as having good knowledge, while 36.7% fell into the poor knowledge category.

**Figure 2:**
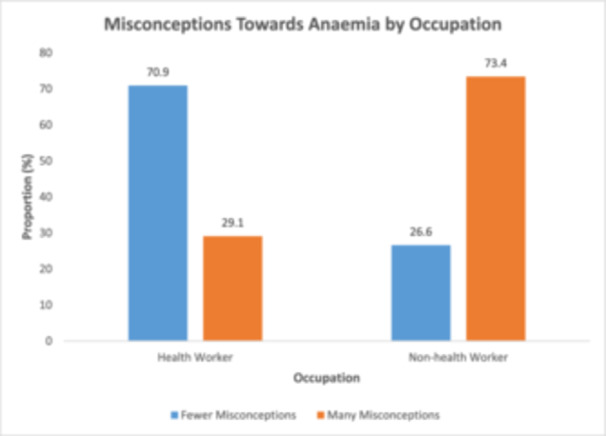
Distribution of misconception categories by occupation.

Figure 2 presents the distribution of misconception categories across occupation groups. Among health workers, 70.9% were classified as having fewer misconceptions, while 29.1% were classified as having many misconceptions. In contrast, 26.6% of non‐health workers were in the fewer misconceptions category, whereas 73.4% were classified as having many misconceptions.

**Figure 3:**
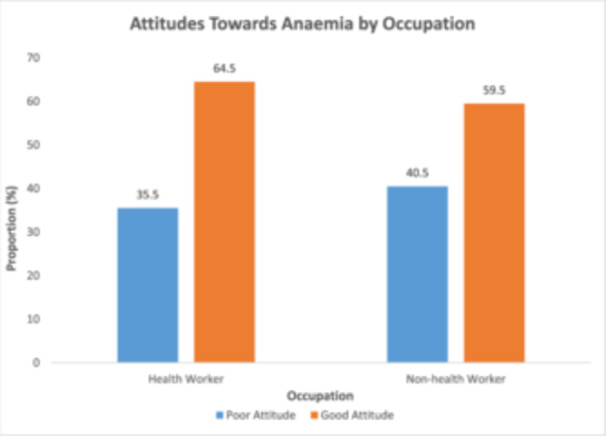
Distribution of attitude categories by occupation.

Figure 3 displays the distribution of attitude categories by occupation. Among health workers, 64.5% were classified as having good attitudes, while 35.5% were classified as having poor attitudes. Among non‐health workers, 59.5% were classified as having good attitudes, with 40.5% falling into the poor attitude category.

### 6.4 Anaemia Prevalence and Predictors

**Figure 4:**
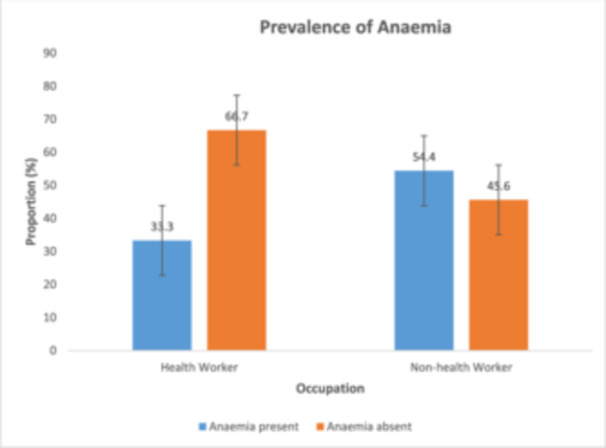
Prevalence of anaemia.

Figure 4 above shows the anaemia prevalence according to occupation. Anaemia was prevalent in 33.3 percent and 54.4 percent of health workers and non‐health workers, respectively.

Table 5a presents the multivariable logistic regression model assessing predictors of anaemia (model *n* = 299; anaemia cases = 133). After adjusting for covariates, occupation showed a notable association with anaemia, with non‐health workers having lower odds (aOR = 0.344, 95% CI: 0.184–0.641, *p* < 0.001). Attitudes/practices category also showed an association (aOR = 0.549, 95% CI: 0.326–0.924, *p* = 0.024). Other variables, including age, sex, marital status, education, region, and history‐related factors, had aORs with confidence intervals crossing 1.0. Model diagnostics indicated adequate fit (Hosmer–Lemeshow *χ*² = 10.590, df = 8, *p* = 0.226) and acceptable multicollinearity (VIF range: 1.060–2.568).

**Table 5a:** Multivariable logistic regression predicting anaemia. Model *n* = 299; Anaemia cases = 133.

Variable	aOR (95% CI)	*p* value
Age (years)		
18–25 (Ref)		
26–30	1.258 (0.618–2.560)	0.527
31–34	1.160 (0.615–2.187)	0.647
Sex		
Male (Ref)		
Female	1.133 (0.625–2.054)	0.680
Marital status		
Single (Ref)		
Married	0.784 (0.467–1.314)	0.355
Educational level		
Non formal (Ref)		
Secondary	1.519 (0.585–3.943)	0.390
Tertiary	1.046 (0.532–2.059)	0.896
Occupation		
Health worker (Ref)		
Non‐health worker	0.344 (0.184–0.641)	< **0.001**
Region of residence		
Northern (Ref)		
Upper East	1.625 (0.833–3.172)	0.155
Upper West	1.262 (0.651–2.448)	0.491
Chronic health status		
Yes (Ref)		
No	0.917 (0.385–2.184)	0.846
History of anaemia in the past 3 years		
Yes (Ref)		
No	0.774 (0.278–2.152)	0.623
Do you take alcohol		
Yes (Ref)		
No	0.823 (0.389–1.743)	0.611
History of blood transfusion		
Yes (Ref)		
No	1.306 (0.516–3.306)	0.573
Have you ever taken iron supplement?		
Yes (Ref)		
No	1.327 (0.715–2.461)	0.370
Knowledge		
Poor (Ref)		
Good	0.710 (0.328–1.539)	0.386
Misconceptions		
Fewer misconceptions (Ref)		
Many misconceptions	0.961 (0.537–1.720)	0.894
Attitudes and practices		
Poor (Ref)		
Good	0.549 (0.326–0.924)	**0.024**

*Note:* Anaemia status was determined by: Hb < 12 g/dL for non‐pregnant women, Hb < 11 g/dL for pregnant women and Hb < 13 g/dL for men. Ref: Referent category. Goodness‐of‐fit: Hosmer–Lemeshow *χ*² = 10.590, df = 8, *p* = 0.226. Multicollinearity**:** VIF range = 1.0603–2.568 (all < 10). Ref: Referent. Bold text indicates statistically significant *p*‐values (*p* < 0.05).

**Figure 5:**
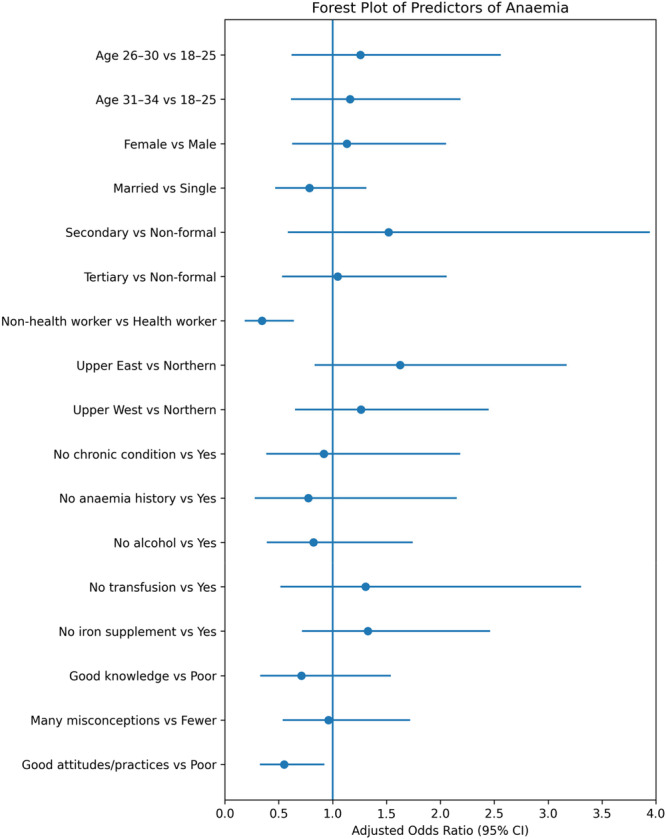
Forest plot of predictors of anaemia. Misconception items were reverse‐coded; higher scores indicate fewer misconceptions.

In the adjusted logistic regression model, being a non‐health worker was associated with lower odds of good anaemia knowledge (OR < 1), indicating reduced likelihood of adequate knowledge compared to health workers. Although anaemia prevalence was higher among non‐health workers, occupation here appears to function as a proxy for educational and socioeconomic status, with health workers demonstrating stronger knowledge profiles. This inverse association is illustrated in the forest plot (Figure 5).

Table 5b displays the linear regression model examining associations between haemoglobin concentration and selected predictors. Occupation showed the largest negative beta coefficient (*β* = −0.188, 95% CI: −1.322–−0.164 to, *p* = 0.012). Other predictors showed beta coefficients close to zero with confidence intervals including null values.

**Table 5b:** Linear regression.

Variable	Std. Error	*β*	95% Confidence Interval		*p* value
			Lower bound	Upper bound	
Age (years)	0.138	0.027	−0.209	0.336	0.647
Gender	0.276	0.027	−0.436	0.650	0.699
Marital status	0.244	−0.112	−0.923	0.039	0.071
Education	0.151	0.025	−0.234	0.359	0.678
Occupation	0.294	−0.188	−1.322	−0.164	**0.012**
Region of residence	0.160	0.101	−0.069	0.561	0.126
Chronic health status	0.410	−0.022	−0.942	0.671	0.741
History of anaemia in past three (3) years	0.480	0.051	−0.678	1.214	0.578
Do you take alcohol?	0.355	−0.111	−1.302	0.096	0.091
Any history of blood transfusion?	0.444	0.033	−0.707	1.041	0.707
Have you ever taken iron/multivitamin supplement?	0.274	−0.105	−0.955	0.125	0.132
Knowledge score	0.063	−0.058	−0.165	0.083	0.517
Misconception score	0.077	−0.072	−0.211	0.092	0.440
Attitudes and Practices score	0.089	−0.068	−0.275	0.074	0.259

### 6.5 Association Between Specific Attitudes and Practices and Anaemia Prevalence

Table 6 examines the association between specific attitudes and practices and anaemia prevalence, stratified by occupation. Among non‐health workers, a significant association was found between preference for vegetables over supplements and anaemia status (*χ*² = 7.299, df = 1, Cramer's *V* = 0.215, *p* = 0.010), suggesting a potential misunderstanding about the sufficiency of diet alone in anaemia management.

**Table 6:** Associations of attitudes and practices of healthcare workers and their patients with anaemia prevalence.

	Health worker (*n* = 141)		Cramer's V	*χ*²	*p* value	Non‐health worker (*n* = 158)		Cramer's *V*	*χ*²	*p* value
	Anaemia status					Anaemia status				
	Present (*n* = 47)	Absent (*n* = 94)				Present (*n* = 86)	Absent (*n* = 72)			
Do you consult a Physician when you have symptoms like dizziness and shortness of breath?			0.108	1.639	0.272			0.095	1.426	0.295
Yes	39 (83.0)	85 (90.4)				57 (66.3)	54 (75.0)			
No	8 (17.0)	9 (9.6)				29 (33.7)	18 (25.0)			
Do you seek health education on anaemia?			0.115	1.855	0.256			0.045	0.327	0.625
Yes	35 (74.5)	79 (84.0)				54 (62.8)	42 (58.3)			
No	12 (25.5)	15 (16.0)				32 (37.2)	30 (41.7)			
I prefer to eat a heavy meal once or twice a day due to financial issues.			0.073	0.759	0.456			0.154	3.731	0.076
Yes	19 (40.4)	31 (33.0)				43 (50.0)	47 (65.3)			
No	28 (59.6)	63 (67.0)				43 (50.0)	25 (34.7)			
Any health worker can prescribe anaemia medication for me			0.019	0.049	> 0.99			0.120	2.293	0.139
Yes	4 (8.5)	7 (7.4)				37 (31.4)	31 (43.1)			
No	43 (91.5)	87 (92.6)				59 (68.6)	41 (56.9)			
Do you think the food you eat has nothing to do with anaemia?			0.032	0.141	0.780			0.007	0.007	> 0.99
Yes	6 (12.8)	10 (10.6)				40 (46.5)	33 (45.8)			
No	41 (87.2)	84 (89.4)				46 (53.5)	39 (54.2)			
I can manage anaemia on my own without the help of a clinician			0.012	0.019	> 0.99			0.007	0.008	> 0.99
Yes	12 (25.5)	23 (24.5)				8 (9.3)	7 (9.7)			
No	35 (74.5)	71 (75.5)				78 (90.7)	65 (90.3)			
I'd rather eat vegetables than take supplements			0.010	0.014	> 0.99			0.215	7.299	**0.010**
Yes	25 (53.2)	51 (54.3)				34 (39.5)	44 (61.1)			
No	22 (46.8)	43 (45.7)				52 (60.5)	28 (38.9)			
Would you participate in pre‐marital counselling and testing before having children?			0.016	0.035	> 0.99			0.110	1.895	0.193
Yes	42 (89.4)	83 (88.3)				69 (80.2)	51 (70.8)			
No	5 (10.6)	11 (11.7)				17 (19.8)	21 (29.2)			
There's no need to follow up on my health once I've been prescribed medication			0.131	2.416	0.180			0.087	1.183	0.313
Yes	6 (12.8)	5 (5.3)				25 (18.8)	16 (9.6)			
No	41 (87.2)	89 (94.7)				108 (81.2)	150(90.4)			
I don't see the importance of sleeping under insecticide treated bed net			0.015	0.033	> 0.99			0.091	1.323	0.327
Yes	6 (12.8)	11 (11.7)				8 (9.3)	11 (15.3)			
No	41 (87.2)	83 (88.3)				78 (90.7)	61 (84.7)			

*Note:* All *χ* ^2^ tests were conducted with degree of freedom (df) = 1. Bold text indicates statistically significant *p*‐values (*p* < 0.05).

Among healthcare workers, no individual attitude or practice variable showed a significant association with anaemia status, although trends in consulting physicians (83.0% of anaemic vs. 90.4% non‐anaemic) and supplement behaviour suggest a potential protective effect. These findings emphasize the importance of reinforcing correct practices, particularly among patients, to complement existing knowledge and reduce anaemia prevalence.

## 7 Discusison

This study offers several distinctive contributions to the literature on anaemia management, primarily due to its focused population and underrepresented geographical setting, which have been largely overlooked in existing research. Unlike most anaemia‐KAP studies that focus solely on patients, this study directly compares knowledge and practices between healthcare providers (HCWs) and their patients in the same clinical settings. This approach reveals critical gaps in provider‐patient communication and identifies where educational interventions are most needed. In addition, the study specifically targets the three northern regions (Northern, Upper East, Upper West), which have Ghana's highest anaemia prevalence [31]. Few studies have examined anaemia‐KAP in this socioeconomically disadvantaged zone, where food insecurity, low literacy, and cultural beliefs heavily influence health behaviours [32]. Data were collected from faith‐based and government hospitals in districts like Bawku and Kpandai, which serve populations with limited healthcare access. These areas face unique challenges, including seasonal malnutrition, malaria endemicity, and displacement due to regional conflicts, factors rarely addressed in urban‐centric anemia studies [33]. The study incorporated verbal consent and interviews for non‐literate patients, ensuring representation of marginalized groups often excluded from research. This is critical in northern Ghana, where 34% of women lack formal education [34].

The findings of this study partially support the hypotheses proposed in the introduction. First, health workers demonstrated higher knowledge and fewer misconceptions about anaemia compared with non‐health workers. Second, although attitudes and practices differed between the groups, the differences were less pronounced than those observed for knowledge and misconceptions. Third, anaemia prevalence was higher among non‐health workers. Finally, the multivariable analysis indicated that attitudes and practices were significantly associated with anaemia status, whereas knowledge and misconception scores were not independently associated with anaemia after adjustment for other variables.

Unlike questionnaire‐only KAP studies, this research integrated haemoglobin measurements to objectively link knowledge gaps to anaemia prevalence. Participants were recruited from diverse facility types (e.g., rural health centres vs. district hospitals), capturing variability in healthcare access. This study fills a critical evidence gap by focusing on a high‐risk, understudied population in a resource‐constrained, culturally distinct setting. Its findings challenge assumptions about anaemia education in low‐literacy contexts and offer a model for regionally adapted public health strategies.

The near‐equal gender distribution (50.8% male, 49.2% female) differs from typical anemia studies that often show female predominance due to reproductive risk factors [35]. However, this aligns with recent facility‐based studies in Ghana demonstrating increased male health‐seeking behavior for chronic conditions [33]. Religious distribution (67.7% Christian, 30.1% Muslim) mirrors northern Ghana's religious demographics but shows slightly higher Christian representation than national surveys (54% Christian, 18% Muslim [34];. This may reflect healthcare utilization patterns, as faith‐based facilities account for 40% of Ghana's health services (MOH, 2022). The low smoking prevalence (2.3%) corresponds with Ghana's 3% adult smoking rate [36], while higher alcohol use (15.7% vs national 8%) may indicate regional consumption patterns or stress‐related coping among health workers, as observed in Nigerian studies [37]. The 46.2% iron supplement usage exceeds the 28% national average for women of reproductive age [38], suggesting better access in clinical populations. These findings highlight the importance of contextualizing anemia interventions within specific sociodemographic frameworks, particularly in regions with unique healthcare access patterns like northern Ghana [39].

In Table 2, the results indicated that healthcare givers generally exhibited a higher level of knowledge about anaemia than their patients did. For instance, 94.3% of healthcare givers accurately identified anaemia as a condition characterized by reduced oxygen‐carrying capacity of the blood whereas only 60.8% of non‐health workers did so. These findings are consistent with other studies [40] which emphasized that healthcare givers are more familiar with anaemia's aetiology and treatment protocol than the general population. However, even among healthcare givers, misconceptions still linger. About 18.4% incorrectly believed that vegetarians are not at risk of Vitamin B12 deficiency anaemia; this suggests that while knowledge about anaemia may be sound, continuous professional education is necessary to address nuanced issues in anaemia management. Meanwhile patients demonstrated lower knowledge and held more misconceptions. Results showed that nearly 30% believed anaemia is a spiritual condition and 8.9% believed it was contagious. These odds were significantly higher than among healthcare givers. According to a similar study [41], cultural misconceptions are known to hinder effective health‐seeking behaviors.

Table 3 demonstrated attitudes and practices towards anaemia prevalence and management, which indicated that most healthcare givers (87.9%) reported to consult a physician for symptoms like dizziness whereas only 70.3% of patients did the same. In addition, 80.9% of healthcare givers sought health education on anaemia compared to 50.8% of patients. This difference suggests a gap in health literacy, which can adversely affect anaemia outcomes. In addition, results stated that the economy influences dietary habits. Over half of the patients (57%) reported eating once or twice due to financial constraints. This supports the findings from a previous study [42], that suggests socio‐economic status is closely linked to anaemia, particularly in low‐income populations where diets often lacks essential nutrients like iron and folate. Additionally, 36.7% of patients believed that any healthcare giver could prescribe anaemia medication while only 7.8% of healthcare givers agree so. This is a clear indication of lack of understanding about medical roles and safe treatment protocols resulting into an inappropriate treatment.

Furthermore, the logistic regression analyses in Table 4 indicated that specific knowledge variables were significantly associated with lower anaemia prevalence. Among healthcare givers and their patients, accurate knowledge that poor nutrition and malaria are contributing factors to anaemia was associated with significantly lower odds of being anaemic. These findings are consistent with studies that link health literacy with improved health outcomes [43]. Awareness of the high risk of anaemia during pregnancy was significantly associated with reduced anaemia prevalence among healthcare givers (AOR = 6.029, *p* = 0.018). This implies that targeted education interventions, which focuses on high‐risk groups, may positively influence personal preventive behaviors. The adjusted model showing non‐health workers having lower odds of anaemia (AOR = 0.344) highlights the limitation of using occupation as a crude proxy for socioeconomic status and highlights the importance of directly measuring socioeconomic determinants such as income, education, and household resources in future studies, as these factors may exert stronger influences on anaemia risk.

In Table 5a, associations of attitudes and practices of healthcare givers and their patients with anaemia prevalence were demonstrated indicating that attitudes and practices influenced anaemia prevalence. Patients preferred consuming vegetables instead of taking supplements was significantly associated with a higher prevalence of anaemia (*p* = 0.010), which potentially reflect a misconception regarding the sufficiency of vegetables alone in meeting iron requirements. Additionally, individuals who were already diagnosed with the condition had more tendency to seek health education on anaemia (*p* = 0.025). Healthcare givers showed no attitudes or practices that was significantly associated with anaemia prevalence. However, increased engagement in medical consultations and consistent adherence to follow up prescription were observed. These findings align with a similar study [44] which highlighted the role of sustained professional practices in mitigating adverse health outcomes.

Despite some level of knowledge about anaemia exhibited in this study, the gaps demonstrated cannot be overemphasized, particularly among patients. These gaps are crucial and can undermine anaemia prevention interventions. In agreement with a previous study [12], the gaps in health knowledge, educational influence, occupation and socio‐economic status shows that cultural factors play major roles in anaemia burden. Overall findings in this study highlights the importance of context‐specific interventions that address both knowledge and behaviour. Amplifying health education programs to increase the awareness of communities to improve access to iron supplements and nutritious foods would actively help in reducing anaemia burden in the northern regions of Ghana.

### 7.1 Theoretical Perspectives on KAP Disparities

The observed KAP disparities between healthcare providers and patients can be understood through multiple theoretical frameworks. The Health Belief Model (HBM) posits that health behaviors are influenced by perceived susceptibility, perceived severity, perceived benefits, and perceived barriers [45]. Our findings suggest that while patients may perceive anaemia as severe (when symptomatic), low perceived susceptibility, particularly among asymptomatic individuals, may reduce motivation for preventive behaviors. Moreover, substantial perceived barriers, particularly economic constraints limiting access to nutritious foods, override knowledge of preventive measures. This explains why knowledge alone does not translate to practice, as evidenced by the 57% of patients reporting financial barriers to adequate nutrition despite moderate awareness of dietary causes.

The Social Determinants of Health (SDH) framework further contextualizes these findings within structural inequities [46]. Poverty, food insecurity, limited education, and inadequate healthcare infrastructure create conditions where knowledge is necessary but insufficient for behavior change. Our data showing that lower household income is independently associated with higher anaemia odds (AOR = 2.34, 95% CI: 1.45–3.78, *p* < 0.001) underscore the primacy of structural determinants over individual knowledge.

Andersen's Behavioral Model of Health Services Use provides insight into healthcare‐seeking patterns [18]. This model emphasizes that service utilization is determined by predisposing factors (demographics, health beliefs), enabling factors (economic resources, insurance, geographic access), and need factors (perceived and evaluated health status). Our finding that only 38% of anaemic patients sought care prior to study enrollment suggests that enabling factors, particularly economic barriers and distance to facilities, limit care access despite recognized need. The model also explains why healthcare providers' superior knowledge does not automatically translate to improved patient outcomes: effective knowledge translation requires not only provider competence but also enabling environments that facilitate patient access and adherence.

These theoretical perspectives collectively suggest that addressing anaemia requires multi‐level interventions; individual‐level education to enhance knowledge and perceived susceptibility, community‐level interventions to address cultural beliefs and social norms, and structural interventions to reduce economic and geographic barriers to nutritious foods and healthcare services.

### 7.2 Knowledge Translation and Communication Gaps

Our finding that healthcare providers possess significantly higher knowledge (94.3% vs. 60.8%, *p* = 0.004) yet patients remain highly anaemic (54.4%) highlights a critical failure in knowledge translation, the process of converting research evidence and clinical knowledge into improved patient outcomes [47]. Several factors may explain this translation gap.

Effective patient education requires not only provider knowledge but also communication skills, cultural competence, and adequate consultation time. In resource‐constrained settings, high patient volumes, limited consultation time (often < 10 min per patient), and language barriers impede effective health education. Healthcare providers may possess biomedical knowledge but lack training in patient‐centered communication techniques that enhance comprehension and retention.

The concept of health literacy; the capacity to obtain, process, and understand basic health information needed to make appropriate health decisions [48], is important. Our data suggest a substantial health literacy gap, with 35% of patients having no formal education. Providers may communicate using medical terminology or abstract concepts that exceed patients' health literacy levels, resulting in poor comprehension despite educational efforts.

Traditional didactic approaches to patient education (one‐way information transfer) are less effective than interactive, participatory methods that assess comprehension, address misconceptions, and tailor messages to individual contexts [49]. Our findings suggest that current patient education practices may rely heavily on didactic approaches without verification of patient understanding or addressing individual barriers.

### 7.3 Socioeconomic Mediation of Dietary Practices

The persistent impact of economic constraints on dietary practices, despite awareness, exemplifies how socioeconomic determinants mediate health behaviors. Among patients who correctly identified iron‐rich foods (72.5%), only 38.6% reported consuming these foods regularly, with 57% citing financial barriers. This finding aligns with Sen's capability approach [50], which posits that health outcomes depend not only on knowledge (a resource) but also on capabilities (the ability to convert resources into outcomes). Economic poverty limits capability: even when individuals possess knowledge of healthy diets, poverty constrains their ability to translate knowledge into practice.

Food insecurity in Northern Ghana is both chronic and seasonal, with the “hunger season” (May‐August) preceding harvest periods [51]. During this period, even middle‐income households reduce dietary diversity and meal frequency. Our finding that consuming < 3 meals/day is associated with 2.8‐fold higher odds of anaemia (AOR = 2.84, *p* < 0.001) underscores how economic constraints directly impact nutritional status, independent of knowledge. This suggests that educational interventions alone are insufficient; effective anaemia reduction requires economic interventions (e.g., cash transfers, food subsidies, agricultural support) to enhance households' purchasing power and food security.

### 7.4 Comparison With Regional and International Evidence

Our findings align with and extend regional evidence on anaemia KAP disparities. The Ghana Demographic and Health Survey (GDHS) 2022 reported 40% national anaemia prevalence among women of reproductive age, with Northern Region prevalence reaching 52% [52] consistent with our patient sample prevalence of 54.4%. However, the GDHS did not assess KAP or compare provider‐patient knowledge, representing a gap our study addresses.

Regional comparisons reveal similar patterns of knowledge‐practice gaps. In Nigeria, Obeagu et al. (2023) [53] found that while 68% of pregnant women in southeastern Nigeria could identify anaemia symptoms, only 42% consumed iron‐rich foods regularly, with financial constraints cited as the primary barrier, remarkably similar to our finding of 57% reporting economic barriers. Their study similarly demonstrated that knowledge scores did not significantly predict anaemia status when controlling for socioeconomic factors, reinforcing our conclusion that knowledge is necessary but insufficient for anaemia prevention in resource‐limited settings.

International comparisons provide additional context. Abu‐Baker et al. (2021) [42] assessed KAP among pregnant women in Jordan, reporting 71% knowledge adequacy but only 48% practice adequacy, with a significant knowledge‐practice gap attributed to cultural beliefs and economic constraints. Notably, their provider‐patient comparison (though limited) showed similar patterns to our study: providers had 95% knowledge adequacy compared to 71% among patients. However, Jordan's higher economic development and universal healthcare coverage resulted in lower anaemia prevalence (32%) compared to Northern Ghana (54.4%), suggesting that health system and economic contexts substantially modify the knowledge‐anaemia relationship.

In Kenya, Kimiywe and Chege [31, 41] found that nutrition education interventions improved knowledge but had minimal impact on anaemia prevalence without concurrent economic support, consistent with our finding that knowledge alone does not predict anaemia status. Their intervention study demonstrated that combining nutrition education with cash transfers or food vouchers produced significantly greater anaemia reduction than education alone, supporting our recommendation for integrated interventions.

Our study's unique contribution lies in the direct provider‐patient comparison using both biochemical and behavioral indicators within the same healthcare settings. While previous studies have assessed either providers or patients separately, few have employed comparative designs that illuminate the translation gap between provider knowledge and patient outcomes. This approach provides actionable insights for intervention design; bridging the provider‐patient gap requires not only enhancing patient knowledge but also improving provider communication skills, addressing health literacy barriers, and implementing structural interventions to reduce economic constraints.

### 7.5 Gender Dimensions of Anaemia Risk

Although not a primary focus of this study, our data reveal important gender dimensions that warrant discussion. Anaemia prevalence was significantly higher among female patients (61.2%) compared to male patients (42.8%; *χ*² = 6.84, *p* = 0.009, Cramer's *V* = 0.21), consistent with known biological and social determinants of gender disparities in anaemia [54].

Women of reproductive age face elevated anaemia risk due to menstrual blood loss, pregnancy‐related demands, and childbirth. Our exclusion of pregnant women likely underestimates the full gender disparity, as pregnancy substantially increases anaemia risk. The higher female prevalence highlights the need for gender‐specific interventions targeting menstrual health, birth spacing, and reproductive health services integration.

Gender inequities in Northern Ghana, including unequal access to education, economic resources, and healthcare decision‐making, may compound biological vulnerabilities [55]. Cultural norms often prioritize male household members in food distribution, particularly for nutrient‐dense foods like meat. Our qualitative observations during data collection (though not systematically captured) suggested that women, particularly in polygamous households, often ate last and consumed smaller portions. This aligns with evidence from other West African contexts showing intra‐household food allocation inequities [56].

Interestingly, our data showed no significant gender difference in knowledge scores among patients (*p* = 0.32), suggesting that knowledge gaps alone do not explain gender disparities. Rather, women's limited economic autonomy and decision‐making power may constrain their ability to translate knowledge into practice. Even when women know which foods prevent anaemia, they may lack financial resources to purchase them or decision‐making authority to prioritize their own nutritional needs.

Future interventions should adopt explicitly gender‐responsive approaches that address the social, economic, and biological factors shaping anaemia risk among women and girls. Economic empowerment strategies, such as cash transfer programs or income‐generating activities targeted at women, can enhance their purchasing power and nutritional autonomy; evidence from conditional cash transfer programs in other settings indicates that resources directed to women are more likely to be spent on household nutrition and children's health. At the household level, engaging men in anaemia prevention education is essential to address inequities in food allocation and decision‐making. Educating male household heads about women's nutritional needs and promoting equitable food distribution can help reduce gender‐based disparities in dietary intake. Integrating anaemia prevention into reproductive health services, including family planning, antenatal, and postnatal care, ensures that women's heightened vulnerability during their reproductive years is adequately addressed, while promoting birth spacing through family planning can reduce the cumulative risk of anaemia associated with closely spaced pregnancies. In addition, addressing menstrual health is critical, particularly through the management of heavy menstrual bleeding and improved access to menstrual hygiene products, which are key contributors to anaemia among adolescent girls and women of reproductive age. Over the long term, reducing anaemia inequities also requires investment in girls' education, as higher educational attainment is associated with improved health literacy, delayed marriage and childbearing, and greater economic opportunities, all of which contribute to lower anaemia risk.

### 7.6 Implications for Ghana's Community‐Based Health Planning and Services (CHPS) Programme

Ghana's Community‐Based Health Planning and Services (CHPS) programme represents the primary healthcare delivery platform in rural and underserved areas, including Northern Ghana [57]. CHPS aims to bring essential health services closer to communities through community health officers (CHOs) and community health volunteers (CHVs). Our findings have several important implications for strengthening anaemia prevention within the CHPS framework.

Currently, anaemia screening within CHPS is limited primarily to antenatal care. Our finding of 54.4% anaemia prevalence among general outpatients suggests the need to integrate routine haemoglobin testing into all CHPS outpatient encounters, not only maternal health services. Point‐of‐care HemoCue devices, as used in this study, are feasible for CHPS settings and could enable early detection and referral.

CHPS community health volunteers conduct home visits and community health education sessions. Our findings suggest that current education efforts are insufficient, as evidenced by persistent misconceptions (29.7% spiritual beliefs, 8.9% contagion beliefs). CHPS education materials should be redesigned using health literacy principles: simplified language, visual aids, culturally relevant examples, and interactive formats that address misconceptions explicitly. Training CHVs in participatory education methods (e.g., demonstration, role‐play, teach‐back techniques) could enhance knowledge retention and translation to practice.

CHPS zones could serve as distribution points for nutrition interventions targeting anaemia. Pilot programs could integrate iron supplementation distribution, food vouchers, or linkages to agricultural extension services promoting kitchen gardens with iron‐rich vegetables. Our finding that economic barriers override knowledge suggests that CHPS‐based nutrition support could have substantial impact.

CHPS staff require training not only in biomedical knowledge (which our data suggest is adequate among healthcare providers) but also in patient‐centered communication, cultural competence, and behavior change counseling. Training curricula should emphasize how to elicit and address patient barriers, tailor messages to health literacy levels, and use motivational interviewing techniques to support behavior change.

CHPS operates through community health committees that engage traditional leaders and community members in health planning. These structures could be leveraged to address cultural misconceptions about anaemia through community dialogues, engaging traditional healers as partners rather than competitors, and using community theater or radio programs to disseminate accurate information in culturally resonant formats.

Strengthening CHPS data systems to routinely capture anaemia prevalence, risk factors, and treatment outcomes would enable monitoring of intervention effectiveness and identification of high‐risk sub‐populations requiring targeted interventions.

### 7.7 Study Limitations

This study has several limitations that should be considered when interpreting findings. The cross‐sectional design precludes causal inference and temporal sequencing. While we identified associations between KAP variables and anaemia status, we cannot determine whether low knowledge preceded anaemia development or whether anaemia experience influenced knowledge. Longitudinal studies are needed to establish causal pathways and temporal relationships.

Our facility‐based sampling may introduce selection bias, as participants were recruited from healthcare facilities and may differ systematically from community members who do not access formal healthcare. This may result in overestimation of knowledge and healthcare‐seeking behaviors, as our sample likely includes more health‐conscious individuals with better access to care. Community‐based sampling would provide more representative estimates.

KAP data relied on self‐reported behaviors, which are subject to social desirability bias, recall bias, and reporting errors. Participants may have overreported socially desirable behaviors (e.g., healthy eating, supplement use) and underreported stigmatized behaviors. Dietary practices, in particular, are subject to recall bias, as participants may not accurately remember consumption frequency. Future studies should employ objective measures (e.g., dietary recalls, food frequency questionnaires validated with biomarkers) and mixed‐methods approaches to triangulate self‐reported data.

Haemoglobin measurements using HemoCue devices in field settings may vary by ±0.5 g/dL due to environmental factors, device variability, and operator technique, despite rigorous quality control procedures. This measurement error may result in misclassification of anaemia status, particularly for individuals near diagnostic thresholds. However, sensitivity analyses using adjusted thresholds showed minimal impact on prevalence estimates, suggesting robust classification.

Data collection occurred over a 4‐month period during the dry season (November 2022 to February 2023). This temporal window may not capture seasonal variations in anaemia prevalence related to malaria transmission (peaks in rainy season) and food security (varies by agricultural cycle). Findings may not be generalizable to other seasons.

The inclusion of only three facilities limits generalizability and statistical power for facility‐level analyses. While facilities were selected to represent regional diversity, findings may not generalize to all healthcare settings in Northern Ghana or other regions of the country.

Despite controlling for multiple sociodemographic, dietary, and health‐related variables, residual confounding from unmeasured factors (e.g., genetic haemoglobinopathies, chronic diseases, medication use) may influence associations. Additionally, we did not assess certain important variables such as parity, pregnancy history, menstrual blood loss, and parasitic infections (beyond self‐reported malaria), which are known risk factors for anaemia. Although questionnaires were translated and back‐translated, subtle linguistic and cultural nuances may have been lost in translation. Some concepts may not have direct equivalents in local languages, potentially affecting response validity.

Despite these limitations, this study provides valuable insights into provider‐patient KAP disparities and their associations with anaemia in Northern Ghana. The integration of biochemical (haemoglobin) and behavioral (KAP) data, comparative design, and multi‐site approach strengthen the study's contributions. Future research should employ longitudinal or mixed‐methods designs to triangulate biochemical data with qualitative insights on dietary behavior, gender norms, cultural beliefs, and health service access barriers.

## 8 Conclusion

This comparative cross‐sectional study reveals significant knowledge, attitude, and practice disparities between healthcare providers and patients regarding anaemia prevention and management in Northern Ghana. While healthcare providers demonstrate superior biomedical knowledge (94.3% vs. 60.8%), this knowledge has not effectively translated to improved patient outcomes, as evidenced by the 54.4% anaemia prevalence among patients. Persistent misconceptions among patients, including beliefs that anaemia is contagious (8.9%) or spiritual (29.7%), highlight critical gaps in health education effectiveness.

Importantly, our findings demonstrate that knowledge alone is insufficient for anaemia prevention. Economic constraints emerged as powerful independent predictors, with participants consuming fewer than three meals daily having 2.8‐fold higher odds of anaemia (AOR = 2.84, *p* < 0.001), and those in the lowest income quartile having 2.3‐fold higher odds (AOR = 2.34, *p* < 0.001). These findings highlight that addressing anaemia requires multi‐level interventions targeting not only individual knowledge but also structural determinants of health.

Addressing anaemia aligns with multiple Sustainable Development Goals, particularly SDG 2 (Zero Hunger) through improved nutrition, SDG 3 (Good Health and Well‐being) through reduced disease burden, and SDG 5 (Gender Equality) through addressing women's disproportionate anaemia risk. Effective anaemia control can contribute to poverty reduction (SDG 1) by improving productivity and economic outcomes, and to quality education (SDG 4) by enhancing children's cognitive development.

Future research should employ longitudinal or mixed‐methods designs to establish causal relationships, assess intervention effectiveness, and understand the complex pathways through which socioeconomic, cultural, and health system factors interact to perpetuate anaemia. Implementation research is needed to identify scalable, cost‐effective strategies for integrating multi‐level interventions into routine health system operations.

Despite study limitations, this research provides critical evidence on the provider‐patient knowledge gap and the primacy of structural determinants over individual knowledge in shaping anaemia outcomes. These insights can inform evidence‐based policy and programmatic decisions to reduce the substantial anaemia burden in Northern Ghana and similar resource‐limited settings across sub‐Saharan Africa.

## 9 Recommendations

Based on our findings, we propose the following evidence‐based recommendations organized into three interrelated domains: the health system, the community, and research.

We recommend the phased integration of routine anaemia screening into primary healthcare services, with prioritized or risk‐based screening for high‐risk populations such as pregnant women, adolescents, and individuals with chronic health conditions. Routine haemoglobin testing should be integrated into all outpatient workflows, extending beyond antenatal care to include all patient groups. The use of point‐of‐care devices such as HemoCue can facilitate feasible screening in resource‐limited settings, and all patients identified with anaemia should receive standardized counseling, appropriate treatment, and structured follow‐up. To support this, pre‐service and in‐service training programs should be implemented to enhance provider competencies in patient‐centered communication, health literacy, and behavior change counseling. Standardized counseling protocols and job aids should guide providers in identifying and addressing individual patient barriers, while mentorship and supportive supervision systems can reinforce effective communication practices. In addition, anaemia prevention indicators, including screening coverage, counseling provision, treatment adherence, and follow‐up completion, should be incorporated into facility‐level quality improvement programs. Strengthening supply chains is also essential to ensure the consistent availability of iron supplements, diagnostic tools, and educational materials through improved forecasting, procurement, and distribution. Finally, health information systems should be enhanced to support routine data collection on anaemia prevalence, risk factors, and intervention coverage, enabling effective monitoring, evaluation, and data‐driven decision‐making.

Based on the identified potential of the Community‐based Health Planning and Services (CHPS) platform highlighted in this study, we recommend strengthening community‐based anaemia education, screening, and prevention initiatives through CHPS compounds and community health officers. At the community level, culturally appropriate education and active engagement are critical for sustainable anaemia prevention. Health education materials should be developed and disseminated in local languages such as Dagbani and Mampruli, using visual aids, storytelling, and culturally resonant approaches. Community dialogues should explicitly address common misconceptions, including spiritual causation and contagion beliefs, with the involvement of religious and traditional leaders. Multiple communication channels such as community radio, market‐day outreach, religious gatherings, and school‐based platforms, should be leveraged to reinforce consistent messaging. Community mobilization efforts should involve partnerships with chiefs, queen mothers, religious leaders, and community‐based organizations to champion anaemia prevention, alongside the establishment of community health committees to support locally tailored interventions. Traditional healers should be engaged as partners and equipped with accurate information to share with their clients. Peer education strategies, including the training of community health volunteers, can support home visits, cooking demonstrations, and group discussions, while women's support groups can promote nutrition, health awareness, and economic empowerment. Addressing underlying economic and food security constraints is also essential; nutrition‐sensitive social protection programs such as cash transfers or food vouchers should target households affected by anaemia, and household food production should be enhanced through kitchen gardens, small‐scale livestock rearing, and linkages to agricultural extension and microfinance services. School‐based interventions should further reinforce these efforts by integrating anaemia prevention into curricula, strengthening school feeding programs with iron‐rich foods, and implementing school‐based screening and treatment initiatives.

Further research is needed to strengthen the evidence base for effective anaemia prevention and control. Longitudinal cohort studies should be conducted to establish causal relationships between changes in knowledge, attitudes, and practices and anaemia outcomes, assess the long‐term sustainability of interventions, and identify critical life‐course windows for prevention. Mixed‐methods research combining quantitative and qualitative approaches is essential to deepen understanding of cultural beliefs, gender dynamics, and household decision‐making processes, as well as to explore barriers and facilitators to behavior change from patient, provider, and community perspectives and assess the acceptability and feasibility of proposed interventions. Implementation research should focus on identifying effective strategies for integrating anaemia interventions into routine health system operations, evaluating scalability and sustainability, and examining contextual factors such as system capacity, community readiness, and political commitment. Economic evaluations, including cost‐effectiveness and cost‐benefit analyses, are necessary to inform efficient resource allocation and policy decisions. In addition, rigorously designed intervention trials using randomized controlled or quasi‐experimental designs, should test multi‐component approaches that combine patient education, provider training, and economic support, while assessing both intermediate outcomes such as KAP changes and ultimate outcomes including haemoglobin levels and anaemia prevalence. Finally, gender and equity‐focused research should explicitly examine intra‐household dynamics, women's empowerment, and how interventions differentially affect socioeconomic groups, with attention to the intersectionality of gender, poverty, education, and other social determinants.

## Author Contributions

**Ellen Afetorgbor Kafui:** data curation, investigation, methodology, resources, writing – review and editing. **Joseph Biluo Ngendal:** data curation, investigation, methodology, resources, writing – review and editing. **Konpuoda David:** data curation, investigation, methodology, resources, writing – review and editing. **Gabriel Owusu:** data curation, investigation, methodology, resources, writing – review and editing. **Joel Karikari Nyarkoh:** data curation, investigation, methodology, resources, writing – review and editing. **Safianu Apalebilah:** data curation, investigation, methodology, resources, writing – review and editing. **Henrietta Eshun:** data curation, investigation, methodology, resources, writing – review and editing. **Ama Frimpomaa Oware:** formal analysis, investigation, methodology, resources, writing – review and editing. **Emmanuel Jingbeja:** data curation, investigation, methodology, resources, writing – review and editing. **David Mawutor Donkor:** data curation, investigation, methodology, resources, writing – review and editing. **Patrick Adu:** conceptualization, project administration, supervision, writing – review and editing. **Joseph Boachie:** conceptualization, project administration, data curation, investigation, methodology, supervision, writing – review and editing.

## Funding

The authors have nothing to report.

## Conflicts of Interest

The authors declare no conflicts of interest.

## Transparency Statement

The lead author David Mawutor Donkor, Joseph Boachie affirms that this manuscript is an honest, accurate, and transparent account of the study being reported; that no important aspects of the study have been omitted; and that any discrepancies from the study as planned (and, if relevant, registered) have been explained.

## Supporting information


**Table S2:** Correlation between Hb and KAP.

## Data Availability

All data will be made available to interested individuals upon request from the corresponding author.
