# Supplementary material for: Knowledge and Practice Gaps in Anaemia Prevention and Management Among Patients and Health Providers in Northern Ghana: A Comparative Cross‐Sectional Study
Source: Health Sci Rep. 2026 Apr 8;9(4):e72307. doi: 10.1002/hsr2.72307 (PMC13062262; doi:10.1002/hsr2.72307)
Supplement: Supplementary file 1 — Table S2: Correlation between Hb and KAP. [file HSR2-9-e72307-s001.docx]

**Supplementary Materials**

**Table S2: Correlation between Hb and KAP**

|  | **Correlation coefficient** | **p-value** |
| --- | --- | --- |
| Knowledge | 0.015 | 0.793 |
| Misconception | 0.033 | 0.576 |
| Attitude | 0.002 | 0.967 |

**Supplementary Appendix A: Final Questionnaire**

**Structured Questionnaire on Knowledge, Attitude, and Practices of Healthcare Providers and Their Patients towards Anaemia Management and Prevention: A Comparative Study in the Northern Region**

**Section 1: Socio-Demographic Information**

1. Age (years): ☐ <20 ☐ 20–25 ☐ 26–30 ☐ 30–34
2. Gender: ☐ Male ☐ Female
3. Marital status: ☐ Single ☐ Married ☐ Other
4. Educational level: ☐ No formal education ☐ Basic ☐ Secondary ☐ Tertiary
5. Occupation: ☐ Health worker ☐ Non-health worker
6. Religion: ☐ Islam ☐ Christianity ☐ Traditional
7. Region of residence: ☐ Northern ☐ Upper East ☐ Upper West
8. Chronic health status: ☐ No ☐ Yes
9. History of anaemia in the past three (3) years: ☐ No ☐ Yes
10. Do you smoke? ☐ Yes ☐ No
11. Do you take alcohol? ☐ Yes ☐ No
12. Any history of blood transfusion? ☐ Yes ☐ No
13. Have you ever taken iron/multivitamin supplements? ☐ Yes ☐ No

**Section 2: General Knowledge Assessment of Participants on Anaemia**

*(Responses: ☐ Yes ☐ No ☐ Not sure)*

1. Blood has a reduced ability to carry oxygen when anaemia is present.
2. Dizziness and weakness may be some symptoms of anaemia.
3. Intake of poor diet and malaria infection are some causes of anaemia.
4. When untreated, anaemia could lead to death.
5. Anaemia could result in impaired development in children.
6. Pregnant women are mostly at risk of anaemia.
7. Vegetarians are mostly at risk of vitamin B12 deficiency anaemia.
8. Excess intake of alcohol leads to anaemia.
9. Iron deficiency can cause anaemia.
10. Intake of non-nutritious substances like clay can cause anaemia.

**Section 3: Assessment of Misconceptions of Participants**

*(Responses: ☐ Yes ☐ No ☐ Not sure)*

1. Anaemia is a spiritual condition.
2. Anaemia is contagious or transmissible by body contact.
3. Sickle cell anaemia affects males more than females.
4. Men are more prone to anaemia compared to women.
5. Consistently eating three meals a day can prevent anaemia.
6. Pregnant women who eat well do not require iron and folic acid supplements.
7. Anaemia is a disorder of the heart.

**Section 4: Prevention and Management of Anaemia**

*(Responses: ☐ Yes ☐ No)*

1. Do you believe anaemia can be prevented?
2. Eating foods rich in iron can help prevent anaemia.
3. Taking iron supplements can help prevent anaemia.
4. Regular medical check-ups help in early detection of anaemia.
5. Deworming can help prevent anaemia.

**Section 5: Attitudes and Practices towards Anaemia Prevention and Management**

*(Responses: ☐ Yes ☐ No)*

1. Do you consult a physician when you have symptoms like dizziness and shortness of breath?
2. Do you seek health education on anaemia?
3. I prefer to eat a heavy meal once or twice a day due to financial issues.
4. Any health worker can prescribe anaemia medication for me.
5. Do you think the food you eat has nothing to do with anaemia?
6. I can manage anaemia on my own without the help of a clinician.
7. I would rather eat vegetables than take supplements.
8. Would you participate in premarital counselling and testing before having children?
9. There is no need to follow up on my health once I have been prescribed medication.
10. I do not see the importance of sleeping under an insecticide-treated bed net.

**Supplementary Appendix B: Sample Items by Domain**

**Knowledge Domain**

- “Blood has a reduced ability to carry oxygen when anaemia is present.”
- “Intake of poor diet and malaria infection are some causes of anaemia.”
- “When untreated, anaemia could lead to death.”

**Misconceptions Domain**

- “Anaemia is a spiritual condition.”
- “Anaemia is contagious or transmissible by body contact.”
- “Men are more prone to anaemia compared to women.”

**Attitudes and Practices Domain**

- “Do you consult a physician when you have symptoms like dizziness and shortness of breath?”
- “Do you seek health education on anaemia?”
- “I prefer to eat a heavy meal once or twice a day due to financial issues.”

**Supplementary Appendix C: Scoring System**

**Item Scoring Rules**

- Knowledge items:
  - Correct response = 1
  - Incorrect / Not sure = 0
- Misconception items:
  - Correct (disagreeing with misconception) = 1
  - Incorrect / Not sure = 0
- Attitudes and Practices items:
  - Preventive or appropriate behaviour = 1
  - Non-preventive or inappropriate behaviour = 0

**Reverse Coding**

- All misconception items were reverse-coded so that higher scores represented fewer misconceptions and more accurate understanding of anaemia.

**Composite Score Calculation**

- Scores for each domain were obtained by summing individual item scores:
  - Knowledge domain total score
  - Misconceptions domain total score
  - Attitudes and Practices domain total score

**Cut-off Determination**

- Domain-specific mean scores were used as cut-off points, consistent with established KAP literature:
  - Knowledge: mean = 9.21 ± 1.00
  - Misconceptions: mean = 6.04 ± 1.30
  - Attitudes and Practices: mean = 5.89 ± 1.12
- Participants scoring at or above the mean were classified as having:
  - Good knowledge
  - Fewer misconceptions
  - More positive attitudes/practices
- Participants scoring below the mean were classified as having:
  - Poor knowledge
  - Many misconceptions
  - Fewer positive attitudes/practices

**Internal Consistency**

- Knowledge domain: Cronbach’s α = 0.91
- Misconceptions domain: Cronbach’s α = 0.70
- Attitudes and Practices domain: Cronbach’s α = 0.80

**Supplementary Appendix D: Correlation between Hb and KAP scores**

Supplementary Table 1 presents the correlation coefficients between haemoglobin level and the knowledge, misconception, and attitude scores. The correlation between haemoglobin level and knowledge score was minimal (r = 0.015, p = 0.793). Similarly, haemoglobin level showed a small correlation with misconception score (r = 0.033, p = 0.576). The correlation between haemoglobin level and attitude score was near zero (r = 0.002, p = 0.967).
